# Characterizing Blood Metabolomics Profiles Associated with Self-Reported Food Intakes in Female Twins

**DOI:** 10.1371/journal.pone.0158568

**Published:** 2016-06-29

**Authors:** Tess Pallister, Amy Jennings, Robert P. Mohney, Darioush Yarand, Massimo Mangino, Aedin Cassidy, Alexander MacGregor, Tim D. Spector, Cristina Menni

**Affiliations:** 1 Department of Twin Research and Genetic Epidemiology, King's College London, London, United Kingdom; 2 Department of Nutrition, Norwich Medical School, University of East Anglia, Norwich, United Kingdom; 3 Metabolon, Inc., Durham, United States of America; Universitat de Lleida-IRBLLEIDA, SPAIN

## Abstract

Using dietary biomarkers in nutritional epidemiological studies may better capture exposure and improve the level at which diet-disease associations can be established and explored. Here, we aimed to identify and evaluate reproducibility of novel biomarkers of reported habitual food intake using targeted and non-targeted metabolomic blood profiling in a large twin cohort. Reported intakes of 71 food groups, determined by FFQ, were assessed against 601 fasting blood metabolites in over 3500 adult female twins from the TwinsUK cohort. For each metabolite, linear regression analysis was undertaken in the discovery group (excluding MZ twin pairs discordant [≥1 SD apart] for food group intake) with each food group as a predictor adjusting for age, batch effects, BMI, family relatedness and multiple testing (1.17x10^-6^ = 0.05/[71 food groups x 601 detected metabolites]). Significant results were then replicated (non-targeted: *P*<0.05; targeted: same direction) in the MZ discordant twin group and results from both analyses meta-analyzed. We identified and replicated 180 significant associations with 39 food groups (*P*<1.17x10^-6^), overall consisting of 106 different metabolites (74 known and 32 unknown), including 73 novel associations. In particular we identified *trans*-4-hydroxyproline as a potential marker of red meat intake (0.075[0.009]; *P* = 1.08x10^-17^), ergothioneine as a marker of mushroom consumption (0.181[0.019]; *P* = 5.93x10^-22^), and three potential markers of fruit consumption (top association: apple and pears): including metabolites derived from gut bacterial transformation of phenolic compounds, 3-phenylpropionate (0.024[0.004]; *P* = 1.24x10^-8^) and indolepropionate (0.026[0.004]; *P* = 2.39x10^-9^), and threitol (0.033[0.003]; *P* = 1.69x10^-21^). With the largest nutritional metabolomics dataset to date, we have identified 73 novel candidate biomarkers of food intake for potential use in nutritional epidemiological studies. We compiled our findings into the DietMetab database (http://www.twinsuk.ac.uk/dietmetab-data/), an online tool to investigate our top associations.

## Introduction

Measurement of dietary intakes in epidemiological settings has traditionally relied on subjective assessment of food intake, which may have resulted in inconsistencies in analyses of associations between specific foods or nutrients and disease endpoints. Although these methods allow us to rank order intakes in large population groups and make comparisons between extreme intake levels, more objective measures, capturing absorption and metabolism *in vivo* are required to further understand the impact of dietary intake and its subsequent metabolism on health.

Nutritional metabolomics involves high-throughput chemical profiling of tissues and biofluids to complement established methods employed in diet- and health-related research and aid biomarker discovery. Recent metabolomics studies have successfully used non-targeted approaches to identify dietary biomarkers in blood in US cohorts, including subjects from the Prostate, Lung, Colorectal, and Ovarian Cancer Screening Trial where 39 potential dietary biomarkers for multiple food groups were identified [1], and subjects from the African Americans in the Atherosclerosis Risk in Communities Study where 39 metabolites were associated with alcohol intake [2] and 48 metabolites to food intakes [2]. Studies using targeted metabolomic approaches have successfully identified significant diet and metabolite associations by examining self-reported dietary intake patterns against serum metabolomic profiles [3–5].

Genetic factors influence metabolic processes, and may account for as much as 81% of the variation in blood levels [6]. There is a complex interplay between genes, diet and metabolism, this is evidenced by mutations causing inborn errors of metabolism which require strict dietary modifications to avoid complications (e.g. phenylketonuria, maple syrup urine disease). Though variation at a number of loci involved in metabolism with less profound single effects are more likely to interact with diet and contribute to complex disease development [7]. Recent dietary intervention trials have investigated the impact of genetic variation of lipid metabolism genes (e.g. cholesteryl ester transfer protein, hepatic lipase gene) on cholesterol levels in response to diets varying in fat content [8,9], though with quite small effects. Using ours (TwinsUK) and the Cooperative health research in the Region of Augsburg (KORA) datasets [10,11], over 400 blood metabolites were associated with 145 metabolic loci, extending the number of potential loci where metabolism, diet and genetics may interact.

Findings of dietary biomarker studies between populations may be difficult to replicate as a result of high inter-individual variability in metabolite levels [12], due to factors including age [13] and genotype [6]. Monozygotic twins, matched for age, sex, early lifestyle factors and baseline genetic sequence, can provide a potential solution to ameliorate issues in reproducibility by acting as controls for one another. Using our twin cohort, we have previously applied this method in one nutri-metabolomic study [14].

Through the use of blood samples profiled by one targeted and another non-targeted metabolomic platforms collected from UK female twins from the TwinsUK cohort, our objective was to identify novel associations between blood metabolites and food intake. We then replicated these associations through the co-twin control method. We supplemented our findings by incorporating results of the GWAS of blood metabolite levels conducted on our dataset previously [6]. Our final aim was to provide the results of our study to the research community through the online DietMetab tool (http://www.twinsuk.ac.uk/dietmetab-data/).

## Materials and Methods

### Ethics Statement

The study was approved by St. Thomas’ Hospital Research Ethics Committee, and all twins provided informed written consent.

### Study population and sample collection

Subjects included in the analysis were female twins enrolled in the TwinsUK registry, a national register of UK adult twins, representative of the UK population [15]. The procedures followed were in accordance with the ethical standards of the responsible institutional or regional committee on human experimentation or in accordance with the Helsinki Declaration of 1975 as revised in 1983. We included 3559 female twins, who completed a 131-item validated FFQ [16] between 1995 and 2007, and had metabolomics and BMI data available within +/- 5 years of completing the diet questionnaire. The 131-item Food Frequency Questionnaire (FFQ) was developed and validated against pre-established nutrient biomarkers for the European Prospective Investigation into Diet and Cancer (EPIC) Norfolk [16]. Quality control, subject exclusion criteria and methods for nutrient determination from FFQ data have been previously described [17]. Briefly, twins reported intake frequencies for the past year of average serving sizes for 131 foods and beverages on a 9-point scale (ranging from never or less than once per month to 6+ times per day). Prior to analysis, intake frequencies were adjusted for total energy intake using the residual method [18] and summed into 71 food groups based on nutrient content, food usage and taste (Table A in S1 File). Data relevant to the present study include BMI and zygosity (determined by methods outlined previously [15]). This study was approved by the St. Thomas’ Hospital Research Ethics committee and all subjects provided informed written consent.

### Metabolomics profiling

Non-targeted mass spectrometry-based metabolomic profiling was conducted by the metabolomics provider Metabolon, Inc. (Durham, NC) on 3559 fasted serum and plasma samples as previously described [13,19]. Further details of the blood metabolomics profiling can be found in Text A in S1 File. The Metabolon platform identified 279 structurally named biochemicals (known metabolites) categorized into the following broad categories: amino acids, carbohydrates, vitamins, lipids, nucleotides, peptides, and xenobiotics. The platform also identified 177 metabolites that were unnamed (unknowns) including 18 of which have since been identified (158 total unknowns). Quality control on the metabolomics dataset was performed as previously described [13,19]. Briefly, raw data were median-normalised by dividing metabolite concentrations by the day median of that metabolite and then inverse-normalised. Metabolites with more than 20% of values missing were excluded to avoid false-positive associations. Minimum run day measures were imputed to the missing values.

A targeted metabolomic assay was also performed in a subset of 858 twins, on samples overlapping with Metabolon profiling, in the TwinsUK study using the Biocrates Absolute IDQ^™^-kit p150 (BIOCRATES Life Sciences, AG, Innsbruck, Austria) as previously described [20,21]. Briefly, the flow injection analysis (FIA) tandem mass spectrometry (MS/MS) method is used to quantify 163 known small molecule metabolites simultaneously by multiple reaction monitoring. Quantification of the metabolites is then achieved by reference to appropriate internal standards.

The Biocrates dataset contains acylcarnitines (C*x*:*y*), hydroxylacylcarnitines [C(OH)*x*:*y*] and dicarboxylacylcarnitines (C*x*:*y*-DC); amino acids; sphingomyelins (SM*x*:*y*) and sphingomyelin-derivatives [SM(OH)*x*:*y*]; and glycerophospholipids (PC). The Biocrates platform measures absolute metabolite value (mM). Prior to analysis, the metabolite serum concentrations were log transformed as these were right-skewed. Eighteen metabolites were overlapping between the Biocrates and Metabolon platforms and were therefore dropped from the targeted analysis, allowing for a total of 145 metabolites analyzed from the Biocrates platform.

### Statistical analysis

Statistical analysis was carried out using Stata version 12.

For each metabolite, random intercept linear regression analysis was undertaken in the first sample (discovery sample) excluding MZ twin pairs discordant (MZ twins with measures one SD apart in food group intake) for each food group. Age, metabolite batch, BMI and family relatedness were included as covariates:

$$\Upsilon_{i}\;=\;\beta_{0}+\beta_{i}{Χ}_{ij}+\gamma_{i}{age}_{ij}+\delta_{i}{BMI}_{ij}+\zeta_{j}+\varepsilon_{ij}$$

where Y_*i*_ is the metabolite and X_*ij*_ is the food group intake of twin *j* from pair *i*, and ζ_*j*_ is the family-specific error component that captures the unobserved heterogeneity or family characteristics.

We adjusted for multiple testing using Bonferroni correction thus giving a significant threshold of 1.17x10^-6^ (0.05/[71 food groups x 601 detected metabolites]). For each significant metabolite-food group association from the discovery sample, the same linear regression analysis was repeated/replicated on the MZ discordant twin pair samples. Associations that (i) passed the 5% level of significance and (ii) were in the same direction as the discovery group (only the latter criteria applied to the targeted platform) were considered replicated. Finally, we combined the results of both analyses using an inverse variance fixed effect meta-analysis that are the reported results. The beta coefficients (β) presented in the results of each linear regression analysis represent the amount of a food group reportedly consumed in servings per week that corresponds to a 1 SD change in the metabolite level.

#### Genotype associations

Genotyping protocols have been outlined previously for the genome-wide association study (GWAS) of the Metabolon metabolomics datasets conducted on ours and the Cooperative health research in the Region of Augsburg (KORA) cohorts [10,11].

To identify if genotyping influenced reported intakes, diet-genotype associations were undertaken on gene variants (50 SNPs) which were associated with blood levels of dietary associated-metabolites (41 metabolites) identified in the former study. In the model, we included genotype (additive) as a predictor of the relevant energy-adjusted food group intake adjusted for age and family relatedness. Statistical significance was defined as 4.76x10^-4^ (0.05/105 tests).

## Results

### Characteristics of the study population

The characteristics of the study population can be found in Table B in S1 File.

### Thirty-nine food groups associate with one-hundred and six metabolites

**Fig 1:**
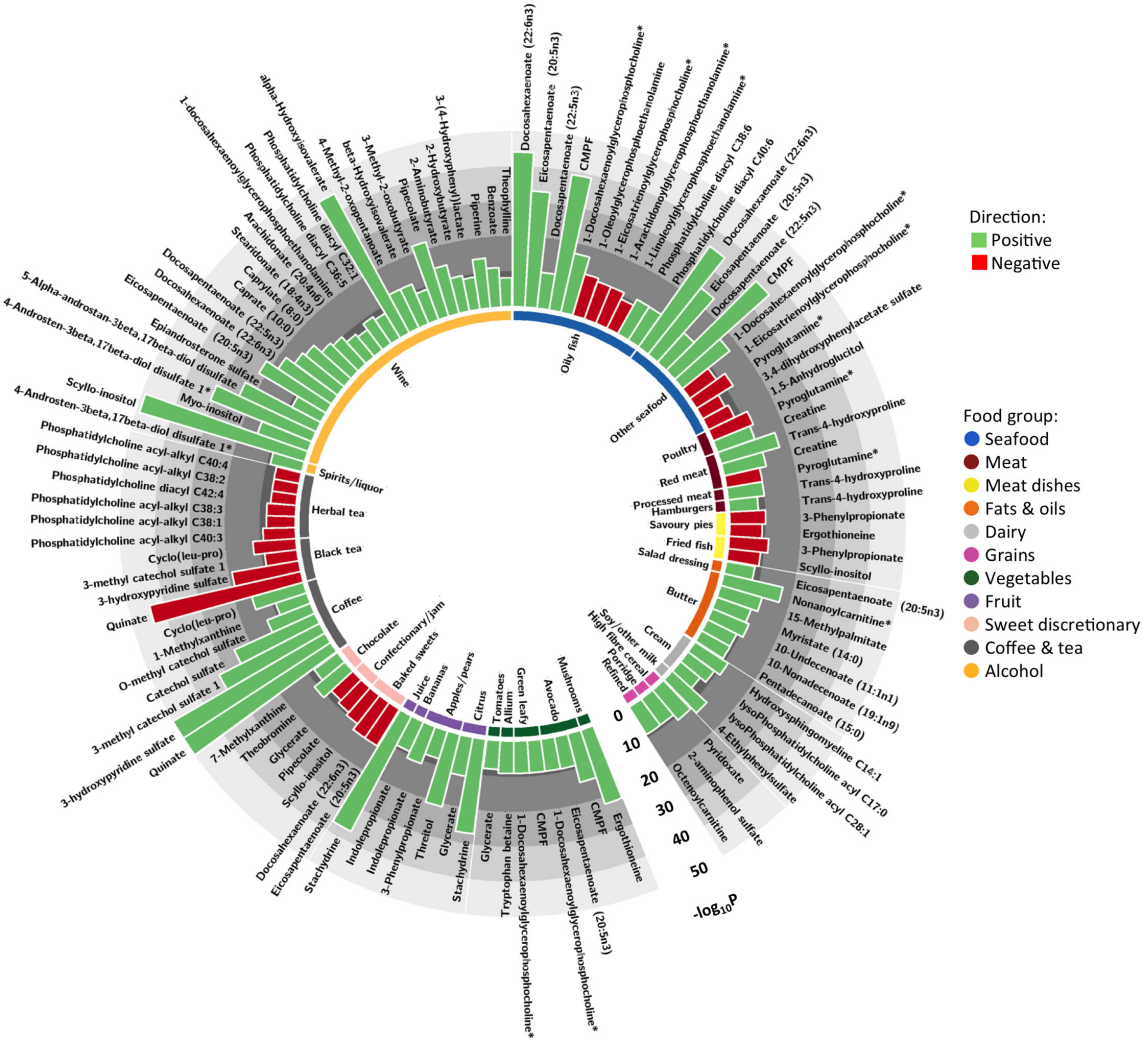
Associations between food group intakes and known metabolites. Associations between food group intakes and known blood metabolites are represented by the circular histogram plot. The histogram bars represent the–log_10_ of the p-value result from the fixed effects meta-analysis and the color of the bars indicates the direction of association: green, positive; red, negative.

**Fig 2:**
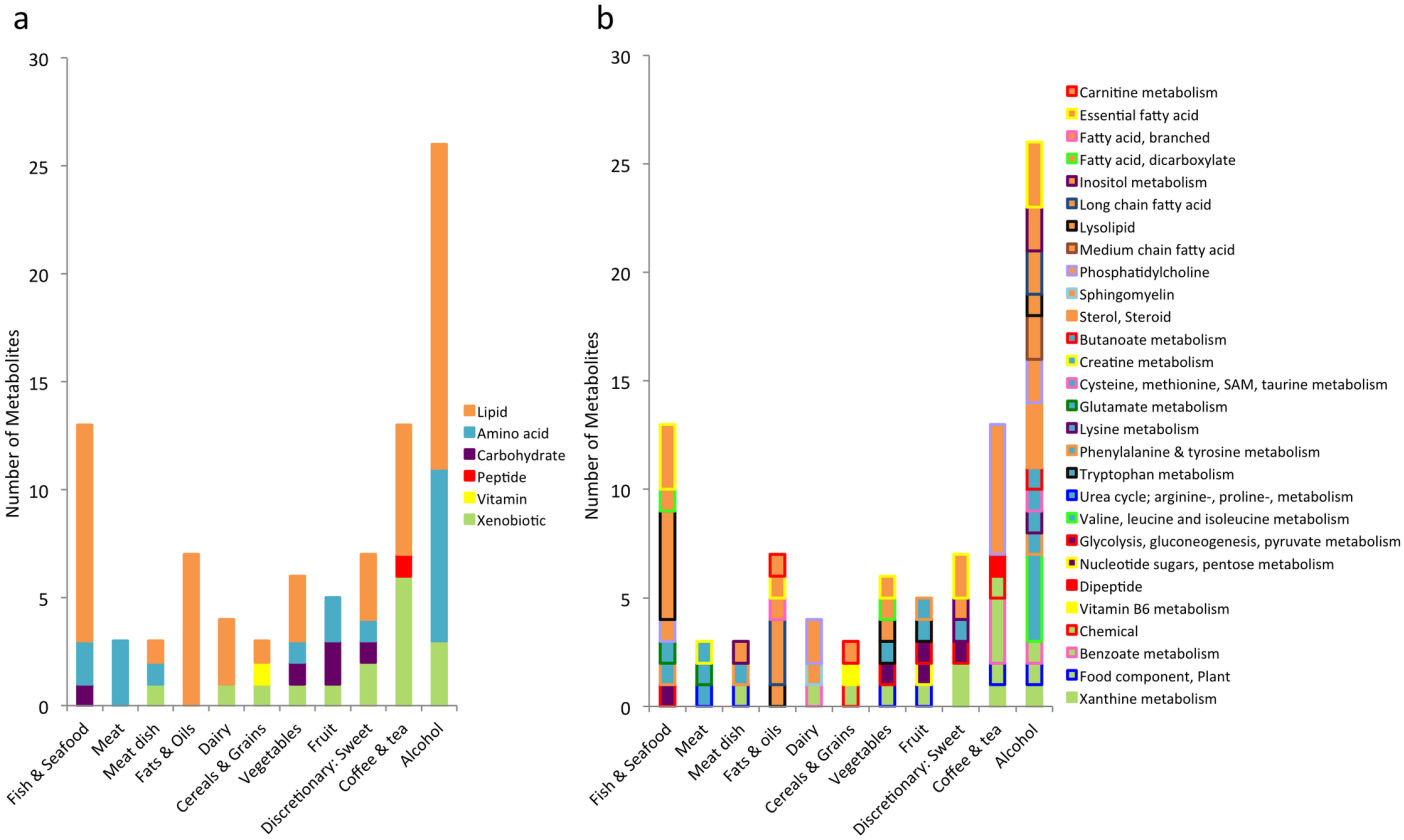
Pathways represented by associated metabolites for general food groups. Fig 2 shows a stacked histogram of the number of associated metabolites representing each superpathway (a) and subpathway (b) by general food group intake.

Of the 601 metabolites measured we found 180 significant associations with 39 food groups after meta-analyzing the discovery and MZ discordant twin groups, overall consisting of 106 different metabolites (Tables C, D and E in S1 File). Of the 106 different metabolites, 74 metabolites were previously been identified (Fig 1) and 32 metabolites are currently unknown. The 74 chemically identified metabolites were attributed to six broad biochemical groups including: 39 lipids, 16 amino acids, 14 xenobiotics, 3 carbohydrates, 1 cofactor/vitamin, and 1 peptide (Fig 2a). Overall the metabolites belonged to 30 different sub-pathways, with metabolites associated to reported alcohol intake being implicated in the most pathways (Fig 2b). To our knowledge, 73 of our known blood metabolite-diet associations have never been identified in large nutritional metabolomics studies before.

The largest number of associations were with reported alcoholic beverage intake, including 42 associations overall (39 wine; 1 spirits/liquors) with 16 associations (of the known metabolites) we believe to be novel; the top association was between wine and scyllo-inositol (meta-analysis result: Beta[SE]: 0.052[0.003]; *p* = 1.47x10^-49^). There were 27 associations (8 novel of the known metabolites) with reported intake of teas and coffee (12 coffee; 9 black tea; 6 herbal tea); the strongest association was between reported coffee intake and the unknown metabolite X-14473 (0.038[0.001]; *p* = 6.12x10^-187^). Twenty-six associations (4 novel) were identified with reported seafood consumption (15 oily fish; 11 other seafood), many of these metabolites are involved in essential fatty acid metabolism; the strongest association found between docosahexaenoate (DHA; 22:6n3) and reported oily fish intake (0.177[0.013]; *p* = 2.09x10^-44^). We identified 9 associations (5 novel) with consumption of meat products (4 meat; 2 poultry; 2 processed meat; 1 beef burgers) which primarily included amino acids; the strongest association was between reported meat intake and trans-4-hydroxyproline (0.075[0.009]; *p* = 1.08x10^-17^), a novel finding. Fourteen metabolites (5 novel) were associated with dairy product consumption (9 butter; 3 cream; 2 low fat milk), the majority of which were lipids, the top association was between low fat milk intake and the unknown metabolite X-21365 (0.076[0.007]; *p* = 9.36x10^-27^). There were 10 associations (3 novel) with reported consumption of grain-rich foods (5 high fibre breakfast cereals; 2 refined bread and grains; 2 porridge; 1 wholemeal bread and grains); the strongest association in this group was between porridge intake and the unknown metabolite X-09789 (0.094[0.008]; *p* = 4.96x10^-33^). There were 13 associations (4 novel) with reported intakes of fruit (6 apple and pears; 3 citrus fruits; 1 bananas; 1 berries; 1 peaches; 1 fruit juices); the top association was between reported fruit juice consumption and stachydrine (0.058[0.005]; *p* = 3.26x10^-37^). There were sixteen associations (8 novel) with reported intakes of vegetables (6 green leafy; 5 avocado; 3 allium; 1 tomatoes; 1 mushrooms), the strongest association was between mushroom intake and ergothioneine (0.181 [0.019]; *p* = 5.93x10^-22^). There were 17 associations (9 novel) with reported intakes of sweet and savoury discretionary foods (5 sweet baked products; 4 savory pies; 4 fried fish; 3 confectionary and jam; 1 savoury snacks); the top association was between consumption of savoury snacks and the unknown metabolite X-11372 (0.051[0.007]; *p* = 3.88x10^-14^). Other notable associations included 2 with reported chocolate intake (1 novel; top association with theobromine: 0.024[0.003]; *p* = 1.34x10^-11^), a novel association between soymilk consumption and 4-ethylphenylsulfate (0.239[0.033]; *p* = 6.05x10^-13^) and unknown metabolite associations with reported consumption of soyfoods (X-11381: -0.108[0.020]; *p* = 5.80x10^-8^) and nuts (X-11315: 0.054[0.005]; *p* = 3.75x10^-25^).

### Gene variants related to metabolites did not influence food intakes

A genome-wide association study to identify genetic variants associated with blood metabolite levels from the non-targeted platform was conducted on our dataset previously [6]. Of these 106 dietary-associated metabolites, 41 contributed to 105 metabolite-SNP associations including 50 unique SNPs in 36 genes (Table F in S1 File). We identified 10 SNP-diet associations at the nominal level (*p*<0.05; Table G in S1 File); the associations did not meet statistical significance following Bonferroni correction (*p*<4.76x10^-4^ = 0.05/105 tests).

## Discussion

In the largest diet-metabolite study so far performed, we identified and replicated in MZ discordant twins 73 novel associations within reported consumption of specific food groups, providing candidate intake biomarkers for future research. A number of these metabolites were previously associated to SNPs, although these did not relate to reported dietary intakes.

### Alcohol consumption

To our knowledge, this is the first study to observe associations between a higher reported wine intake and increased levels of metabolites of branched-chain amino acids (BCAA; valine, leucine and isoleucine and their metabolites, 3-methyl-2-oxobutyrate and 4-methyl-2-oxopentanoate) and medium-chain fatty acid metabolism (caprate and caprylate). The former have previously been shown to be elevated in subjects with type 2 diabetes or impaired fasting glucose in TwinsUK [19] and positively correlated with BMI [22]. Interestingly, 3-methyl-2-oxobutyrate was found to be the strongest predictor of impaired fasting glucose [19]. Elevated levels of BCAA catabolites may signal mitochondrial dysfunction that results in impaired mitochondrial oxidation of glucose and lipids. Binge drinking has been found to induce insulin resistance [23] though the impact of moderate long-term alcohol consumption is not clear, and these associations have identified a potential pathway involved.

We also confirm elevated levels of metabolites associated with higher reported alcohol intake from a previous metabolomics study [2]; in particular, circulating levels of the amino acid alpha-hydroxyisovalerate, the inositol metabolite lipid scyllo-inositol and sulphated steroids derived from dehydroepiandrosterone metabolism (DHEA; 5-alpha-androstan-3beta,17beta-diol disulfate, 4-androsten-3beta,17beta-diol disulfate 1, 5-alpha-androstan-3beta,17beta-diol disulfate and epiandrosterone sulfate) [2]. Interestingly, alpha-hydroxyisovalerate associates to a variant in the *HAO2* gene (rs12141041) encoding long-chain L-2-hydroxy acid oxidase 2 which has been shown to be involved in blood pressure regulation in animal models [24]. Scyllo-inositol is associated with a variant in the *SLC5A11* gene (rs4787294) which encodes a myo- and scyllo-inositol transporting sodium-dependent glucose transporter. Markers in the *SLC5A11* gene have been implicated in systemic lupus erythematosus (SLE) susceptibility. Individuals with SLE have presented with lower levels of amyloid beta (Aβ) in cerebrospinal fluid [25], on which scyllo-inositol has demonstrated protective effects *in vivo* [26]. Blood 4-androsten-3beta,17beta-diol disulfate 1 and 5-alpha-androstan-3beta,17beta-diol disulfate were associated to variants in *SULT2A1* (rs2547231 and rs296396), a gene which catalyzes the sulfation of a wide range of steroids and bile acids. Recently, ethanol feeding in rats significantly increased liver and intestinal expression of *SULT2A1* [27], implicating a direct role for this gene in modulating this association.

### Seafood consumption

Higher reported fish and seafood consumption was uniquely associated with lower levels of pro-inflammatory lysolipids derived from essential fatty acid (EFA) metabolism (1-arachidonoylglycerophosphoethanolamine, 1-eicosatrienoylglycerophosphocholine, 1-linoleoylglycerophosphoethanolamine, 1-oleoylglycerophosphoethanolamine). Lysolipids help form or contribute to forming the cellular lipid bilayer. When cleaved by lipoprotein-associated phospholipase A2, lysolipids form free lysophosphatidylcholines involved in inflammatory processes and may contribute to artherosclerotic plaque inflammation [28]. In line with previous reported associations, higher reported intakes of oily fish and other seafood were associated with higher levels of the furan fatty acid, 3-carboxy-4-methyl-5-propyl-2-furanpropanoate (CMPF) and the EFA docosahexaenoate (DHA) [1,29]. Interestingly, levels of EFA-derived lysolipids and DHA were associated to variants in *FADS1* (rs174538, rs174556, rs968567 and rs174535), which encodes a delta-5 desaturase enzyme [30]. A recent meta-analysis of gene-diet interaction studies found that two of the variants in *FADS1* associated with EFA metabolites in our study (rs174538 and rs174548) modulated gene-dietary-derived EFA associations [31].

### Meat consumption

Metabolite super-pathways affected by meat consumption were primarily amino acids, in particular creatine, trans-4-hydroxyproline and pyroglutamine. We report a novel association between reported red meat intake and trans-4-hydroxyproline, an amino acid that forms part of the collagen structure with elevated levels observed following gelatin consumption [32]. We also identified a unique association between reported intakes of red meat and poultry and circulating creatine levels, of which red meat is the major source and vegetarians have lower blood levels [33]. Blood levels of creatine have been negatively associated with insulin sensitivity [34], lower in liver steatotic versus NASH patients [35], and elevated in dilated cardiomyopathy [36]. Blood levels of creatine associate with a variant in the mitochondrial *CPS1* (rs715). CPS1 converts ammonia into urea as the first enzyme of the urea cycle, and expression of this gene has been identified as a candidate marker of NAFLD [37]. Moreover, we find that circulating levels of pyroglutamine, a metabolite with little known biological function, associate with reported seafood and meat intakes. Lower levels in blood of pyroglutamine have previously been associated with chicken intake [1]. Blood levels of pyroglutamine were linked to a variant in *SLC6A13* (rs11613331) which encodes GAT2 a gamma-aminobutyric acid and betaine transporter. Polymorphisms in *SLC6A13* have previously been associated to renal function [38,39]. Together, these metabolites could be promising biomarkers of animal derived protein intake in future epidemiological studies.

### Dairy consumption

Increased reported consumption of cream (double and clotted cream) was uniquely associated with higher levels of two lysophosphatidylcholines (lysoPhosphatidylcholine acyl C17:0 and C28:1) and hydroxysphingomyeline C14:1. In a metabolomics study of the EPIC-Potsdam cohort, a diet pattern high in butter and high-fat dairy products and low margarine intake was strongly associated with lysoPhosphatidylcholine acyl C17:0 [5]. The saturated fatty acid heptadecanoic acid (C17:0) has been confirmed recently as a biomarker of milk fat intake in a dairy intervention trial and is believed to be quite specific to milk fat due to its formation by ruminal bacteria [40].

Reported butter consumption was associated uniquely with six lipids, primarily fatty acids. We identified three novel associations with butter intake: nonanoylcarnitine, an ester of carnitine with pelargonic acid (C9); 10-nonadecenoate (19:1n9), a monounsaturate of nonadecenoate (19:0); and myristate (14:0), contained in most animal and vegetable fats, with higher concentrations in plasma associated with heart failure [41] and lower levels associated with type 2 diabetes or impaired fasting glucose in TwinsUK [19]. Blood levels of nonanoylcarnitine were associated with a variant in *ACADL* (rs3738934), a gene important for lipid oxidation. Interestingly, *ACADL* expression is reduced in mouse liver and adipocytes under high fat feeding, a process blocked by gene-transfer induced overexpression of *Il-15* [42]. We also confirmed top associations with butter intake from previous nutritional metabolomic studies: pentadecanoate (15:0), 10-undecenoate (11:1n1) and 15-methylpalmitate [1,29].

### Grain-rich product consumption

We report a novel significant association between higher reported intakes of high fibre breakfast cereals and increased levels of the vitamin B6 metabolite, pyridoxate. Pyridoxate is an essential nutrient, coenzyme for synthesis of amino acids, neurotransmitters (serotonin, norepinephrine), sphingolipids, and aminolevulinic acid. Elevated levels of pyridoxate have previously been associated with higher reported intakes of vitamins/supplements and other fruits (including plums, apricots, peaches, prunes, raisins, grapes, pineapple), and high scores on the Healthy Eating Index [1]. Breakfast cereals are typically fortified with B vitamins including vitamin B6 which may have accounted for elevated blood levels of the metabolite. Interestingly, individuals with higher reported porridge consumption had higher levels of the recently identified metabolite, 2-aminophenol sulfate (X-12253) and elevated urinary levels of this metabolite have previously been reported in consumers of wholegrain rye bread versus refined wheat bread in a cross-over intervention study [43]. 2-aminophenol sulfate is characterized as a benzoxazinoid metabolite, benzoxazinoids are found in whole grains and evidence suggests that they are well absorbed from these sources [44]. Subjects reporting higher intakes of refined grain products had increased levels of octenoylcarnitine, an acylcarnitine formed from mitochondrial beta-oxidation. Interestingly, in a previous study, octenoylcarnitine levels were reduced in 33 coeliac disease patients on a long-term gluten free diet [45].

### Fruit consumption

Reported intake of apples and pears were uniquely associated with threitol, a sugar alcohol, and two amino acids formed by gut bacteria: indolepropionate (also with bananas) [46,47] and 3-phenylpropionate [46]. 3-phenylpropionate is also formed by gut bacterial transformation of polyphenolic compounds [48,49] and has recently been shown to be formed directly from gut microbial catabolism of apple proanthocyanidins when incubated with human gut bacteria [50]. Lower circulating indolepropionate has previously been associated with reported intakes of eggs and red meat in a US population [1], suggesting that either these subjects consumed less fruit-derived proanthocyanidins or the bacterial catabolism of the polyphenolic compounds is compromised with higher intakes of animal proteins. In other metabolomic studies, higher levels of indolepropionate have been associated with better insulin sensitivity [34], and lower levels associated with lower muscle mass in elderly subjects [51]. Interestingly, SNPs within medium-chain acyl-CoA synthetase (MACS) genes were identified for both 3-phenylpropionate (*ACSM5*, rs11647589) and indolepropionate (*ACSM2A*, rs1394678). MACS catalyse the ligation of medium-chain fatty acids with CoA to produce medium-chain acyl-CoA, however members of MACS also conjugate glycine with xenobiotic-derived benzoic acid derivatives [52]. Along with 3-phenylpropionate, benzoic acid is a product of gut microbial degradation of apple and cranberry phenolic compounds [50], suggesting the genotypic association with these metabolites may be mediated by their correlation to products of this process. A variant in the *ACSM2* gene has previously been associated with metabolic syndrome phenotypes; however, this relationship may be primarily related to this gene’s role in lipid metabolism [53]. We confirm previous results between reported fruit juice consumption and stachydrine (also known as proline betaine), a plant component found in high concentrations in citrus fruits, confirming results from other smaller studies [1,29].

### Vegetable consumption

We identified ergothioneine to be most strongly associated with reported consumption of mushrooms—a novel finding. Ergothioneine is a thiol compound with demonstrated *in vivo* protection against lipid peroxidation [54] found in high concentrations in specialty mushrooms, in particular oyster and king bolete, and in lower amounts in oat bran and beans [55]. Recent data supports the notion that ergothioneine may prevent against vascular dysfunction [56]. Positive associations between reported consumption of both green/leafy vegetables and avocado with seafood derived-metabolites, CMPF and 1-docosahexaenoylglycerophosphocholine, may have occurred due to strongly correlated intakes, a similar issue encountered by other authors [2,57,58].

### Tea and coffee consumption

A higher reported intake of herbal tea was associated with a reduction of hepatic-derived long-chain phosphatidylcholine acyl-alkyls [59]. In the EPIC Potsdam cohort, a dietary pattern with high reported intake of red meat and fish and low intake of whole-grain bread and tea was found to correlate with lower levels of phosphatidylcholine diacyl (including phosphatidylcholine diacyl C42:4). In the same cohort, levels of these phosphatidylcholines have been implicated in diabetes risk [60]. The origin of these associations are currently unclear.

We confirm previous associations between increased levels of metabolites of caffeine and coffee components with higher reported coffee and caffeine consumption [2,57]. Many metabolites associated with coffee intake were inversely associated with black tea consumption suggesting that individuals who reported consuming more tea habitually consumed less coffee. We did identify one novel association between higher reported coffee consumption and increased levels of O-methyl catechol sulfate. One of our top associations with coffee is 1-methylxanthine, a product of caffeine metabolism that was associated with a variant in *NAT2* (rs4921914), liver NAT2 acetylates caffeine metabolites [61]. Recently a polymorphism in *NAT2* has been found to modulate the association between black tea consumption and SLE risk [62].

### Sweet & Savoury discretionary food consumption

A number of associations between foods where less consumption is encouraged (‘discretionary’; including sweets and jams, sweet baked products, fried potatoes and fish, crisps and savoury pies) appear to lack biological plausibility and are novel, suggesting these associations may be reflecting reduced intakes of other foods (i.e. vegetables, fruit, fish and wine). For example, higher reported intakes of sweet baked products (including cookies, cakes, pies and pastries) were associated with lower levels of the fatty acids, DHA and EPA (markers of fish intake) and scyllo-inositol (marker of wine consumption). Moreover, strong inverse associations were found between reported intakes of fried fish and savoury pies and levels of the amino acid 3-phenylpropionate derived from gut microbial catabolism of proanthocyanidins found in fruits [50]. Accurate reporting of these foods may have been compromised by the biases of self-reporting [63], a limitation of ours and other similar dietary datasets.

### Other notable associations

We identified a novel association between higher reported soymilk consumption, even though intake levels were low as expected in a UK population, and increased levels of 4-ethylphenylsulfate; an association between this metabolite and tofu consumption has been reported previously [1]. 4-ethylphenylsulfate is a uremic toxin produced by gut bacteria which has been shown to induce anxiety-like behaviours in rats as a result of increases in gut permeability, although evidence suggests that it is alleviated by supplementation with the probiotic *Bacteroides fragilis*, which corrects gut permeability [64]. A potential mechanism for this association may be the characteristically high saponin content of soybeans. Soybean-derived saponins increase intestinal mucosal cell permeability *in vitro* [65*]*, and in Atlantic Salmon [66], which promotes the intestinal absorption of poorly absorbed substances [65] like 4-ethylphenylsulfate.

We identified a novel positive association between reported chocolate intake and 7-methylxanthine and confirmed a previous association with theobromine, a bitter alkaloid from the cocoa plant and marker of cocoa consumption [67]. 7-methylxanthine is a methylated purine formed from the metabolism of methylxanthines (caffeine, theophylline and theobromine) [68]; it is a purine component in urinary calculi and may therefore influence the development of urolithiasis [69].

### Notable unknown metabolite associations

Though work is ongoing, we do not currently know the chemical identity of the 32 metabolites, which may become important dietary biomarkers in future studies. Notably, we identified an association between higher reported intake of fried food (fried fish and savoury snacks (including potato crisps)) and increased levels of the metabolite X-11372. Moreover, we also identified another potential marker of red and processed meat consumption; metabolite X-11381 associated with a polymorphism in *SLC16A9* (rs12356193) which encodes a carnitine efflux transporter [70]. Interestingly, variants in this gene have also been associated with serum uric acid concentrations [71] and susceptibility to gout in renal overload [72]. The unknown metabolite, X-09789, associated with reported porridge intake was associated with a variant in *SLC51A* (rs7642243). The *SLC51A* gene (also known as *OST-alpha*) is a component of the Ost-alpha/Ost-beta complex, which has a vital role in intestinal bile acid transport from the enterocytes into portal blood [73]. Interestingly, oats contain the soluble fibre beta-glucan which is known to lower cholesterol levels [74] through sequestering bile acids in the gut and lowering bile acid reabsorption [75]. The unknown metabolite, X-11315 associated with reported intakes of 13 different foods (top association: apple and pears; negative associations with discretionary foods) was associated with a variant in the *SLC6A20* (rs4327428). The SLC6A20 gene encodes a transporter that has the ability to transport the amino acid proline, suggesting this unknown metabolite is structurally similar to proline. Polymorphisms in this gene have been associated with susceptibility of Type 2 diabetes in white-European and Chinese populations [58].

### Strengths and Limitations

Despite replicating many findings from previous dietary metabolomics studies, our study had a number of potential limitations. Firstly, our population was only female and therefore, although unlikely, our results may not apply to men. Ideally, we would have replicated our novel associations from the meta-analysis in a separate population, which would have further strengthened our findings. Though using MZ discordant twins as our validation sample was advantageous by providing strongly matched controls of the same age, sex and baseline genetic sequence. Reassuringly, we confirm top associations from similar studies, establishing the quality of our data. Our study, being cross-sectional, does not allow us to attribute cause and effect to our findings; however, many of our associations are supported by biological mechanisms. By relying on the FFQ as our means of estimating dietary intakes, we encounter issues due to the nature of self-reported data [63]. However, the majority of our associations are biologically plausible and we replicated some results from dietary intervention studies and findings from two US samples [1,2,29]. We recognize a minority of our associations may have been a result of type I errors due to the correlation of reported intakes, an issue we appeared to have encountered with reported intakes of sweet and savoury discretionary foods (sweets and jams, sweet baked products, fried fish and savoury pies), black tea and vegetables. Moreover, FFQ data is categorical and therefore does not allow us to precisely quantify the effect of food intake on metabolite levels. Not having longitudinal data on metabolite levels we were unable to evaluate the stability of these metabolites over time, though a previous study suggested metabolite levels are generally stable for at least 7 years [76]. By using very stringent cut-offs for multiple testing we believe we minimised the number of spurious associations. Though, with our very stringent cut-offs it is also likely we experienced a number of false-negatives. We benefitted from having genotypic profiling available on a large sample of our population (with replication from the KORA study), allowing us to speculate on the potential metabolic and disease relationships of the dietary associations, which potentially adds validity as genotyping is unbiased.

## Conclusions

By using one of the largest and comprehensive datasets of its kind, we have identified 180 self-reported food intake associations (73 novel) with blood metabolites using stringent cutoffs for multiple testing, adjusting for confounders and replicating our associations using the co-twin control method. Future studies should aim to undertake dietary interventions trials to confirm our findings, adequately determine mechanisms for associations and quantify the effect of food intake on metabolite levels. The findings of our study can be visualized using the DietMetab search tool (http://www.twinsuk.ac.uk/dietmetab-data/).

## Supporting Information

S1 File Supporting Information. (XLSX)
